# Comment on: “Experimental ischaemic stroke induces transient cardiac athrophy” by Veltkamp *et al*.

**DOI:** 10.1002/jcsm.12638

**Published:** 2020-12-19

**Authors:** Nadja Scherbakov, Wolfram Doehner

**Affiliations:** ^1^ Berlin‐Brandenburg Center for Regenerative Therapies (BCRT) Charité‐Universitätsmedizin Berlin Berlin Germany; ^2^ German Center for Cardiovascular Research (DZHK) Berlin Germany; ^3^ Department of Cardiology Charité‐Universitätsmedizin Berlin Berlin Germany; ^4^ Center for Stroke Research Berlin (CSB) Charité‐Universitätsmedizin Berlin Berlin Germany

In the recent issue of the *Journal of Cachexia Sarcopenia and Muscle*, we read with great interest the article by Veltkamp *et al*. “Experimental ischaemic stroke induces transient cardiac atrophy”^1^ in which the authors investigate cardiac function in a murine model of ischaemic stroke. With a global prevalence over 18 million cases in 2013, ischaemic stroke is one of the leading diseases worldwide. Patients with stroke develop a wide range of complications, including physical disability in basic activities of daily living, hemiparesis, aphasia, depression, body weight loss and cachexia.^2^, ^3^, ^4^ However, myocardial injury characterized by structural or functional impairment with or without troponin elevation, as well as electrocardiographic and echocardiographic changes, is frequently observed in patients with acute stroke and is one of the early post‐stroke complications with high predictive value for disease outcome. In a clinical setting, the diagnosis of stroke‐induced cardiac injury is confirmed, among others, by exclusion of previous structural heart diseases in the patients' medical history.

In the present study, the authors investigated cardiac function in mice with experimentally induced acute cerebral ischaemia by middle cerebral artery occlusion (MCAO). Animal models have been well‐established to study pathophysiology of wide range of medical conditions including ischaemic stroke, cancer, cachexia and heart failure.^5^, ^6^, ^7^, ^8^ The authors observed a reduction of left ventricular ejection fraction (LVEF), fractional shortening and heart rate as early as 1 h after induction of cerebral ischaemia, and cardiac dysfunction still persisted after 2 weeks of acute stroke. These changes were accompanied by elevation of high‐sensitive Troponin T levels during the first 3 days. In addition, a decrease of heart weight and a cardiomyocyte cross‐sectional diameter by up to 15% with partial restoration after 2 weeks has been observed. The findings of this study indicated cardiac muscle wasting induced by impaired catecholamine homeostasis that was confirmed by elevated norepinephrine and transcript levels of atrogin‐1 and murf‐1, which are E3 ubiquitin ligases involved in the degradation of skeletal muscle proteins.^9^ The current study provides important insides into the mechanisms of cardiac damage triggered by cerebral ischaemia and contributes to the understanding of the brain‐heart syndrome.^10^ In line with presented results, elevated high‐sensitive cardiac troponin T levels have been frequently observed after stroke.^10^, ^11^ In the present study, the authors additionally investigated echocardiographic parameters, such as LVEF and fractional shortening. The changes of these parameters were observed only in mice with MCAO lasting not 30 min but 60 min. The question is whether an ultrafast intravenous thrombolysis or endovascular treatment in patients with acute ischaemic stroke would have the same protective effect on cardiac function because in a short‐term MCAO occlusion (30 min) in mice, no structural and functional changes of the heart were observed. The majority of clinical studies investigating the effect of reperfusion therapy in acute stroke assess as an endpoint the functional outcome but not the myocardial function of patients with stroke.^12^ Therefore, the longitudinal prospective clinical studies, investigating cardiac function before and after acute stroke, are warranted.

## Conflict of interest

The authors declare that they have no conflict of interests and certify that they comply with the ethical guidelines for authorship and publishing in the Journal of Cachexia, Sarcopenia and Muscle.^13^
